# Effect of Receptive Music Therapy Using the 'U-Shaped Sequence' on Anxiety in Patients Undergoing Radiotherapy for Gynecological and Breast Cancers

**DOI:** 10.7759/cureus.85986

**Published:** 2025-06-14

**Authors:** Sara El Azzouzi, Nabila Sellal, Saloua Lemrabett, Fadila Bousgheiri, Najdi Adil, Mohamed El Hfid

**Affiliations:** 1 Department of Radiation Therapy, University Hospital Mohammed VI, Tangier, MAR; 2 Department of Radiation Therapy, Abdelmalek Essaadi University, Tangier, MAR; 3 Department of Epidemiology and Public Health, University Hospital Mohammed VI, Tangier, MAR; 4 Department of Epidemiology, Faculty of Medicine and Pharmacy of Tangier, Abdelmalek Essaadi University, Tangier, MAR; 5 Department of Radiotherapy, Faculty of Medicine and Pharmacy of Tangier, Abdelmalek Essaadi University, Tangier, MAR

**Keywords:** anxiety, gynecological and breast cancers, music therapy, radiotherapy, u-shaped sequence

## Abstract

Introduction

Anxiety is a common experience among cancer patients, occurring at various stages of the oncological care continuum from the initial diagnosis to treatment and post-therapeutic follow-up. To investigate the therapeutic potential of receptive music therapy using the U-shaped sequence in the context of radiotherapy, we conducted a study to evaluate the effect on patient-reported anxiety during treatment.

Methods

Thirty female patients undergoing radiotherapy were assigned to either an intervention or a control group. The intervention group participated in a 20-minute receptive music therapy session during their third radiotherapy treatment. Each patient selected music of her choice, which was played through a Bluetooth speaker. The control group received standard care without music therapy. Anxiety levels were assessed in both groups during the third radiotherapy session using the validated Arabic version of the State-Trait Anxiety Inventory (STAI, Form Y-1), administered both before and after the session.

Results

In the control group, anxiety scores increased significantly following the radiotherapy session (p<0.000). In contrast, the intervention group exhibited a significant reduction in anxiety scores (p<0.000). Baseline anxiety levels prior to treatment were similar between the two groups, with no statistically significant difference (p=0.075). However, post-treatment anxiety levels were significantly lower in the music therapy group compared to the control group (p<0.000).

Conclusion

This study confirms the high prevalence of anxiety among women undergoing radiotherapy for gynecological and breast cancers and demonstrates that receptive music therapy following a U-shaped sequence significantly reduces anxiety during treatment.

## Introduction

Worldwide, 41% of all newly diagnosed female cancer patients are affected by either breast or gynecological cancers [1]. Cancer can cause profound emotional, physical, and social distress, severely disrupting the patient’s emotional, psychological, and personal balance. Anxiety affects over 50% of cancer patients, with approximately 30% experiencing chronic anxiety [2]. According to the fifth edition of the Diagnostic and Statistical Manual of Mental Disorders (DSM-5) by the American Psychiatric Association, anxiety is defined as an emotion characterized by excessive apprehension, persistent worry, and physiological arousal in response to a perceived threat, whether real or anticipated. It may present with physical symptoms (e.g., tachycardia, elevated blood pressure, muscle tension, sleep disturbances) and cognitive symptoms (e.g., rumination, difficulty concentrating) [3].

Cancer treatment commonly includes surgery, radiotherapy, chemotherapy, and other systemic therapies. Radiotherapy may be administered before or after surgery, either as a standalone treatment or in combination with chemotherapy or other systemic modalities. Receiving radiotherapy (RT) is associated with anxiety in at least one-third of patients without a prior anxiety disorder [4,5]. The prevalence of anxiety at the beginning of RT has been estimated at 35.9%, decreasing to 18.4% post-treatment [6]. Anxiety is linked to poor adherence to cancer treatments, which can compromise both recovery and quality of life [7,8]. In recent years, various non-pharmacological interventions have demonstrated positive effects in reducing treatment-related anxiety [9], among which is music therapy (MT).

According to Munro and Mount [10], music therapy is defined as "the intentional use of the properties and potential of music and its impact on the human being." There are two main types of music therapy: active music therapy, which involves instrumental or vocal participation, and receptive music therapy, which involves passive listening to live or recorded music selected by either the clinician or the patient [11]. Receptive music therapy is more accessible and easier to implement in hospital settings.

A Cochrane meta-analysis [12] highlighted the beneficial effects of music therapy on anxiety, pain, fatigue, mood, and overall quality of life in cancer patients. Additionally, music has shown modest positive effects on heart rate, respiratory rate, and blood pressure. Several studies in the literature have reported on the use of music therapy to reduce anxiety levels in patients undergoing radiotherapy [1,5,6,13,14]. To our knowledge, no such study has been conducted in Morocco.

This study, conducted in the Radiotherapy Department of the Mohamed VI University Hospital in Tangier, northern Morocco, aimed to assess the impact of music therapy, based on songs selected by patients with gynecological or breast cancer, on anxiety during radiotherapy sessions.

## Materials and methods

Eligibility criteria

Eligible participants were female patients under the age of 60 undergoing radiotherapy, fluent in Arabic, and who had not taken any sedative medication within the previous 24 hours. Patients with visual or auditory impairments, or with diagnosed psychiatric or neurological disorders, were excluded from the study.

A total of 60 voluntary patients who met the inclusion criteria were recruited and randomly assigned into two groups. One group received music therapy (MT) and listened to songs of their choice according to the validated standardized “U-shaped sequence” protocol, while the control group did not receive any MT.

Ethics approval and consent

This prospective observational study involved the application of non-pharmacological, non-invasive music therapy as a supportive care intervention for patients undergoing radiotherapy for gynecological or breast cancer. The intervention was implemented as part of routine supportive care and did not modify the patients' standard clinical management. According to institutional policy, and given the minimal risk nature of the intervention, formal approval from the institutional review board (IRB) was not required. All patients were informed about the study and provided verbal informed consent prior to participation, in accordance with the principles of the Declaration of Helsinki.

Data collection tools

Data were collected using the validated Arabic version of the standard State-Trait Anxiety Inventory (STAI-Form Y1). Clinical data were retrieved from electronic medical records.

The Arabic Version of the State-Trait Anxiety Inventory

The STAI, developed by Spielberger et al. [15], is a validated self-report instrument designed to assess anxiety levels. It includes 40 items rated on a four-point Likert scale. The instrument distinguishes between two dimensions of anxiety: trait anxiety (a stable personality characteristic) and state anxiety (a temporary emotional response to a specific situation). The Arabic version of the questionnaire was validated by Bahammam [16]. The STAI-Form Y1 (STAI-S) consists of 20 items that ask participants to indicate how they feel ''right now,'' selecting the most appropriate response. In our study, the STAI-S was used to measure anxiety levels at the moment the questionnaire was administered, and scoring was performed according to the recommended guidelines.

Music intervention procedure

Individual 20-minute playlists were created based on each patient's musical preferences and recorded on MP3 players labeled with their names. The music was played in the radiotherapy treatment room using Bluetooth speakers. MT was conducted during the third radiotherapy session, following the validated “U-shaped music sequence” protocol [17,11].

The "U-shaped music sequence" receptive MT technique is a structured method aimed at inducing deep relaxation. It is based on a specific musical progression lasting 20-25 minutes, composed of six musical pieces of approximately 3 minutes and 30 seconds each, played seamlessly with cross-fades, and arranged according to a precise protocol: M1 (Tempo 80-95 bpm / Instruments: 10-20), M2 (Tempo 60-80 bpm / Instruments: 5-10), M3 (Tempo 40-60 bpm / Instruments: 2-5), M4 (Tempo 30-40 bpm / Instruments: 1-3), M5 (variation of M2) (Tempo 40-60 bpm / Instruments: 2-5), and M6 (variation of M1) (Tempo 60-80 bpm / Instruments: 5-10). The U-sequence includes three phases: a descending phase, a low/stable phase, and an ascending phase.

Control group

Patients in the control group received no information about the MT intervention during the study period to avoid any potential bias.

Statistical analysis

Data were analyzed using IBM SPSS Statistics for Windows, Version 25.0 (Released 2017; IBM Corp., Armonk, New York, United States). The Kolmogorov-Smirnov test was used to assess the normality of variable distribution. Quantitative variables were expressed as mean±standard deviation (SD) or median with interquartile range (IQR), depending on the results of the normality test. Qualitative variables were presented as frequencies and percentages. Intra-group analysis was performed using the Wilcoxon signed-rank test to compare STAI scores before and after the radiotherapy session in both the MT and control groups.

To compare the evolution of anxiety between the two groups, the difference (Δ) between post-treatment and pre-treatment STAI scores was calculated for each participant. An inter-group comparison was then conducted using the Mann-Whitney U test. To assess associations between anxiety evolution and participants' characteristics, the Kruskal-Wallis and Mann-Whitney tests were used. A p-value of <0.05 was considered statistically significant.

## Results

The mean age of the patients included in the study was 48.04 ± 7.659 years. The most represented age group was between 46 and 59 years, accounting for 63.3% of participants. The majority of patients were married (55%) and illiterate (58.33%). Regarding diagnosis, 50% of the women had breast cancer, 30% had cervical cancer, and 20% had endometrial cancer.

The group not exposed to music therapy (MT) showed a significant increase in their anxiety scores (mean±SD=4.80±2.524; median [IQR]=5 [4]; p<0.000), whereas the group exposed to MT experienced a marked reduction in anxiety scores (mean±SD= -28.17±1.367; median [IQR]= -28 [4]; p<0.000). Anxiety levels were comparable between the two groups prior to radiotherapy, with no statistically significant difference (p = 0.075). However, following the radiotherapy session, anxiety levels were significantly lower among patients exposed to MT compared to those who were not (p < 0.000) (Table 1). A comparison of the baseline characteristics between the experimental and control groups is provided in Table 2.

**Table 1:** STAI scores before and after radiotherapy in both groups. Δ: Difference in STAI score (after – before) for each group, expressed as median and IQR. STAI: State-Trait Anxiety Inventory.

	Before	After	p (test)	Δ
Experimental group, N=30	67.67±3.94	39.50±4.06	0.000 (t=27.12)	-28 [2]
Control group, N=30	63.74±5.93	68.27±4.50	0.000 (t=–5.30)	5 [4]
P	0.075	0.000 (t=–29.95)		0.000

**Table 2:** Participant characteristics in the experimental and control groups.

Characteristic	Experimental group, N (%)	Control group, N (%)
Age (years)		
35–45	12 (40.0)	10 (33.3)
46–59	18 (60.0)	20 (66.7)
Marital status		
Single	4 (13.3)	5 (16.7)
Married	18 (60.0)	15 (50.0)
Divorced	4 (13.3)	4 (13.3)
Widowed	4 (13.3)	6 (20.0)
Education level		
Illiterate	16 (53.3)	19 (63.3)
Primary	14 (46.7)	9 (30.0)
Secondary	-	2 (6.7)
University	-	-
Cancer site		
Breast	14 (46.7)	16 (53.3)
Cervix	8 (26.7)	10 (33.3)
Endometrium	8 (26.7)	4 (13.3)

Due to the limited sample size, some nominal variable categories had no observations, reducing the number of analyzable categories to fewer than two for certain variables. As a result, the Mann-Whitney test was used instead of the Kruskal-Wallis test to ensure better statistical power. No statistically significant differences were observed in the distribution of anxiety score changes (Δ scores) across categories of age, marital status, educational attainment, or tumor site between the experimental and control groups (p>0.05) (Table 3).

**Table 3:** Distribution of Δ (STAI score difference after/before MT) in both groups. STAI: State-Trait Anxiety Inventory; MT: music therapy.

Characteristic	Experimental group (mean±SD / median [IQR])	Control group (mean±SD / median [IQR])	Test statistic	p value
Age			U=267	0.491
35–45 years	-27.92±1.505 / -28 [3]	5.20±3.084 / 5 [4]		
46–59 years	-28.33±1.283 / -28 [4]	4.60±2.257 / 4.5 [1]		
Education level			U=918	0.457
Illiterate	-28.19±1.471 / -28 [3]	4.58±2.317 / 4 [1]		
Primary	-	-		
Secondary	-28.14±1.292 / -28 [2]	5.22±3.270 / 5 [6]		
University	-	-		
Marital status			H=6.09	0.111
Single	-27.50±1.732 / -27 [3]	4.80 ± 2.049 / 5 [4]		
Married	-28.22±1.517 / -28 [3]	5.20±2.624 / 5 [4]		
Divorced	-28±0.816 / -28 [2]	6.25±2.062 / 6.5 [4]		
Widowed	-28.75±0.500 / -29 [1]	2.83±2.229 / 4 [4]		
Tumor location			H=2.06	0.488
Breast	-28.43±1.555 / -28 [3]	4.63±2.527 / 4.5 [4]		
Cervix	-28.50±0.535 / -28.5 [1]	5.20±3.084 / 5 [4]		
Endometrium	-27.38±1.408 / -27 [3]	4.50 ± 0.577 / 4.5 [1]		

## Discussion

In our study, we observed a significant decrease in STAI-Y1 scores among patients exposed to MT (M= -28.17; 95% CI [-28.68, -27.66]; p<0.000). In contrast, patients who did not receive MT showed an increase in anxiety levels after treatment (M=4.80; 95% CI [-5.74, -3.86]; p<0.000), compared to their pre-radiotherapy levels, despite comparable baseline anxiety scores in both groups. These results are consistent with previous studies suggesting that music therapy can significantly reduce anxiety in patients undergoing radiotherapy compared to those who do not receive such intervention [9,13,14,18-22].

However, some studies have not observed a statistically significant reduction in anxiety following music therapy. For instance, although patients who received a music-based intervention reported a decrease in anxiety levels, the reduction was not statistically significant when compared to the control group that did not receive music therapy. These findings suggest that, while music therapy may have a calming effect, the differences observed between groups were insufficient to reach statistical significance, thus questioning the effectiveness of this approach in certain contexts [5,23].

Music is closely tied to personal experience, which is why it is essential for patients to select the music they wish to listen to themselves [11,24]. The effectiveness of music therapy may be influenced by the type of music chosen. Patients who listen to music of their own preference often experience a greater reduction in anxiety [13,20,21]. Previous studies have demonstrated that listening to music arranged in U-shaped sequences induces deep relaxation, highlighting its positive impact on patients' well-being and overall sense of calm [17,11].

**Figure 1:**
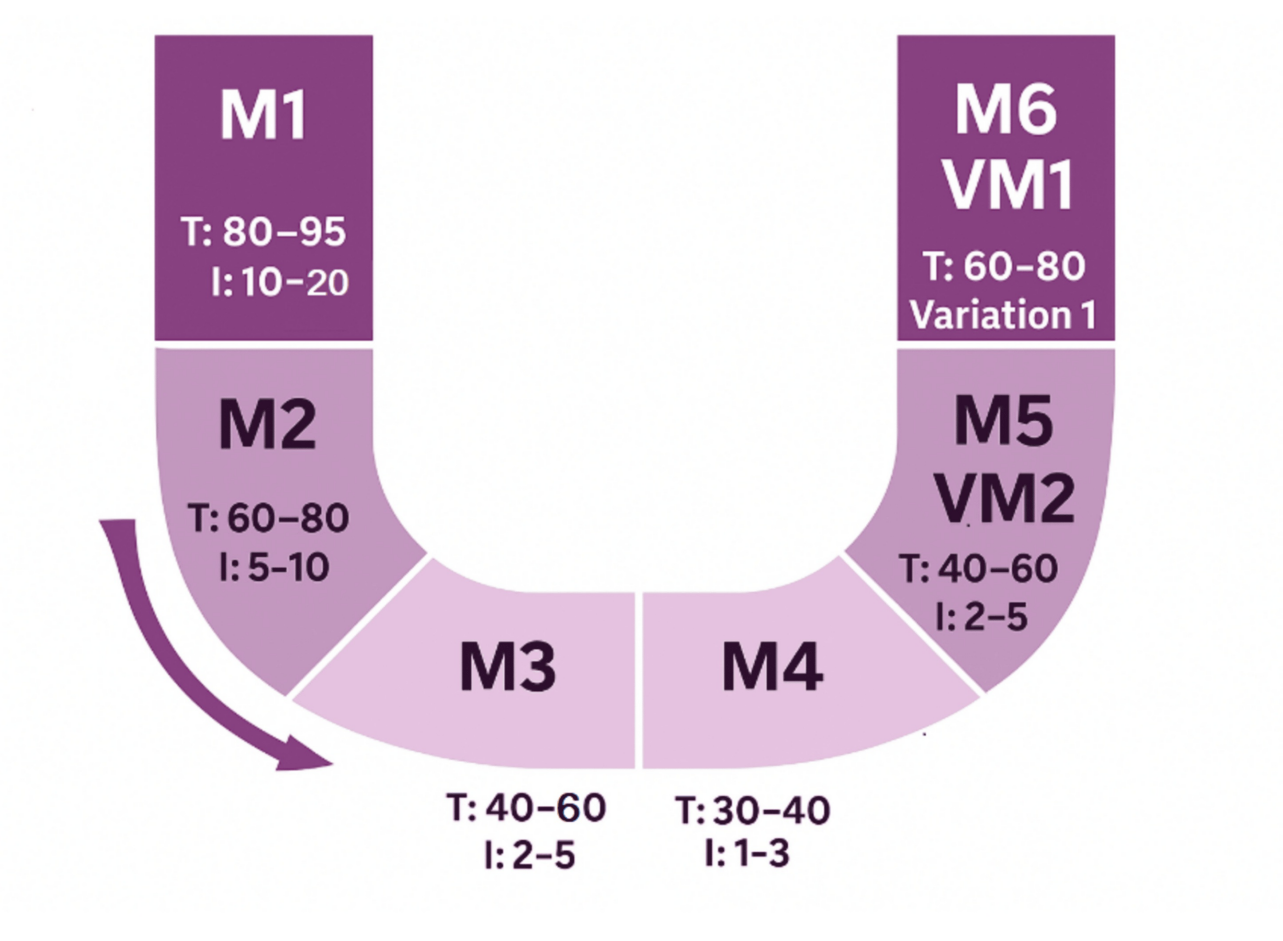
Structural framework of the ''U-Shaped'' receptive MT session. MT: music therapy.

The "U-shaped" music sequence consists of three distinct phases (Figure 1) [17,11]: Descending phase (Induction - Tracks 1 & 2): The goal of this initial phase is to gradually guide the patient into a relaxed state. To achieve this, the selected music pieces progressively decrease in tempo, instrumentation, sound frequency, and volume, thereby creating a soothing atmosphere that promotes the release of physical and mental tension. Bottom phase (Deep Relaxation - Tracks 3 & 4): At the lowest point of the "U," the patient reaches a state of deep relaxation. The music in this phase is characterized by slow tempos, minimal instrumentation, and soft, gentle tones that foster calmness and serenity. Ascending phase (Reactivation - Tracks 5 & 6): The final phase aims to gradually return the patient to a state of alertness. This is achieved by progressively increasing the tempo, instrumental complexity, and volume of the music, thereby facilitating a smooth transition back to wakefulness while maintaining the benefits of the prior relaxation. This technique is particularly used to reduce anxiety, stress, and pain and to promote sleep. It is comparable to other relaxation methods such as sophrology or hypnoanalgesia.

The U-shaped music sequences were developed and proposed by the radiotherapy team based on each patient’s musical preferences, as part of an individualized and personalized care approach [17]. Psychological factors also appear to play a role in reducing anxiety symptoms. A unique bond often develops between the patient and the medical team. The receptive music therapy technique, by eliciting emotions, allows them to be safely expressed within a therapeutic framework. As Munro notes, the emotions conveyed through music can evoke memories and “touch, comfort, and heal, while fostering a sense of being understood or stimulated” [10].

Although numerous studies have explored the role of music in alleviating anxiety during radiotherapy, none have specifically addressed the use of U-shaped sequences as a music therapy strategy. The goal of this research is to deepen the understanding of the effects of musical structure and characteristics in order to better integrate them into therapeutic settings. Regarding the use of music listening during radiotherapy, future studies involving larger sample sizes should consider prolonged exposure to auditory stimuli; for example, by introducing music listening sessions several days before and continuing throughout the course of radiotherapy.

Limitations

The primary limitation of our study is that we assessed the effects of music during a single radiotherapy session. However, radiotherapy is typically administered over 15 to 28 daily sessions across a three- to six-week period. The impact of music may differ if evaluated over a prolonged duration involving multiple treatment sessions. A second limitation lies in the absence of a certified music therapist within our clinical team. Nevertheless, some staff members had a solid understanding of music, which allowed for the integration of this approach in a thoughtful and patient-sensitive manner, despite the lack of formal music therapy expertise.

## Conclusions

Anxiety is a common and clinically significant concern among women initiating radiotherapy for gynecologic and breast cancers. The STAI, given its ease of administration, remains a valuable tool for assessing anxiety in clinical radiotherapy settings. In this study, receptive music therapy using U-shaped musical sequences was associated with a significant reduction in anxiety levels. These findings suggest that music therapy may serve as a simple, cost-effective, and non-pharmacological approach to improve psychological outcomes in oncology care. Further prospective studies are warranted to confirm its effectiveness and guide the development of standardized protocols for music-based interventions in radiotherapy.

## References

[1] Alcântara-Silva TR, de Freitas-Junior R, Freitas NM (2018). Music therapy reduces radiotherapy-induced fatigue in patients with breast or gynecological cancer: a randomized trial. Integr Cancer Ther.

[2] Marrs JA (2006). Stress, fears, and phobias: the impact of anxiety. Clin J Oncol Nurs.

[3] American Psychiatric Association (2022). DSM-5-TR Diagnostic and Statistical Manual of Mental Disorders, Revised Text. https://www.elsevier-masson.fr/dsm-5-tr-manuel-diagnostique-et-statistique-des-troubles-mentaux-texte-revise-9782294781353.html.

[4] Hess CB, Chen AM (2014). Measuring psychosocial functioning in the radiation oncology clinic: a systematic review. Psychooncology.

[5] O'steen L, Lockney NA, Morris CG, Johnson-Mallard V, Pereira D, Amdur RJ (2021). A prospective randomized trial of the influence of music on anxiety in patients starting radiation therapy for cancer. Int J Radiat Oncol Biol Phys.

[6] Hernández Blázquez M, Cruzado JA (2016). A longitudinal study on anxiety, depressive and adjustment disorder, suicide ideation and symptoms of emotional distress in patients with cancer undergoing radiotherapy. J Psychosom Res.

[7] Niedzwiedz CL, Knifton L, Robb KA, Katikireddi SV, Smith DJ (2019). Depression and anxiety among people living with and beyond cancer: a growing clinical and research priority. BMC Cancer.

[8] Pitman A, Suleman S, Hyde N, Hodgkiss A (2018). Depression and anxiety in patients with cancer. BMJ.

[9] Zang L, Cheng C, Zhou Y, Liu X (2022). Music therapy effect on anxiety reduction among patients with cancer: a meta-analysis. Front Psychol.

[10] Munro S, Mount B (1978). Music therapy in palliative care. Can Med Assoc J.

[11] Jourt-Pineau C, Guétin S, Védrine L, Le Moulec S, Poirier JM, Ceccaldi B (2013). Effets de la musicothérapie sur la douleur et l'anxiété des patients atteints de cancer hospitalisés et/ou suivis en service d'oncologie: une étude de faisabilité. Douleurs.

[12] Bradt J, Dileo C, Magill L, Teague A (2016). Music interventions for improving psychological and physical outcomes in cancer patients. Cochrane Database Syst Rev.

[13] Rossetti A, Chadha M, Torres BN, Lee JK, Hylton D, Loewy JV, Harrison LB (2017). The impact of music therapy on anxiety in cancer patients undergoing simulation for radiation therapy. Int J Radiat Oncol Biol Phys.

[14] Chen LC, Wang TF, Shih YN, Wu LJ (2013). Fifteen-minute music intervention reduces pre-radiotherapy anxiety in oncology patients. Eur J Oncol Nurs.

[15] Spielberger CD, Gorsuch R, Lushene RE, Vagg PR (1983). Manuel pour l’inventaire d’anxiété état-trait (STAI).

[16] Bahammam MA (2016). Validity and reliability of an Arabic version of the state-trait anxiety inventory in a Saudi dental setting. Saudi Med J.

[17] Guétin S, Giniès P, Siou DK (2012). The effects of music intervention in the management of chronic pain: a single-blind, randomized, controlled trial. Clin J Pain.

[18] Zeppegno P, Krengli M, Ferrante D (2021). Psychotherapy with music intervention improves anxiety, depression and the redox status in breast cancer patients undergoing radiotherapy: a randomized controlled clinical trial. Cancers (Basel).

[19] Önsüz Ü, Can G (2025). Music therapy in various physical and mental conditions and its effects on cancer patients receiving radiotherapy. Psikiyatride Güncel Yaklaşımlar.

[20] Raglio A, Oddone E, Meaglia I (2021). Conventional and algorithmic music listening before radiotherapy treatment: a randomized controlled pilot study. Brain Sci.

[21] Hanedan Uslu G (2017). Influence of music therapy on the state of anxiety during radiotherapy. Turk Onkoloji Dergisi.

[22] Nardone V, Vinciguerra C, Correale P, Guida C, Tini P, Reginelli A, Cappabianca S (2020). Music therapy and radiation oncology: state of art and future directions. Complement Ther Clin Pract.

[23] Smith M, Casey L, Johnson D, Gwede C, Riggin OZ (2001). Music as a therapeutic intervention for anxiety in patients receiving radiation therapy. Oncol Nurs Forum.

[24] Verdeau-Pailles J (1991). Aspects of psychotherapies. Music therapy and its specificity (Article in French). Encephale.

